# Understanding psychiatrists' knowledge, attitudes and experiences in identifying and supporting their patients on the autism spectrum: online survey

**DOI:** 10.1192/bjo.2019.12

**Published:** 2019-04-05

**Authors:** Laura Crane, Ian Davidson, Rachel Prosser, Elizabeth Pellicano

**Affiliations:** Associate Professor, Centre for Research in Autism and Education, UCL Institute of Education, UK; RCPsych Autism Champion and Consultant Psychiatrist, Royal College of Psychiatrists; and ASD Service, Cheshire and Wirral Partnership NHS Foundation Trust, UK; Undergraduate Placement Student, Centre for Research in Autism and Education, UCL Institute of Education, UK; Professor of Educational Studies, Department of Educational Studies, Macquarie University, Australia

**Keywords:** Autism, psychiatrist, diagnosis, identification, self-efficacy

## Abstract

**Background:**

Psychiatrists play a critical role in identifying and supporting their patients on the autism spectrum in the UK, yet little is known about their knowledge, attitudes and experiences in this regard.

**Aims:**

To understand psychiatrists' experiences of working with autistic individuals, their confidence in making diagnostic/management decisions and the factors that affect such decisions.

**Method:**

A total of 172 psychiatrists took part in an online self-report survey.

**Results:**

Most psychiatrists reported receiving useful training on autism and were knowledgeable about the condition, particularly those with a personal connection to autism. Higher confidence in working with autistic patients was linked to greater levels of autism knowledge, experience and training. Several systemic and autism-specific factors were highlighted by psychiatrists, which were felt to challenge their ability to provide effective care and support for their patients on the autism spectrum.

**Conclusions:**

Psychiatrists' views corroborated previous research with the autism community, highlighting the need to co-design services that are accessible, respectful and person-centred.

**Declaration of interest:**

I.D. is the Royal College of Psychiatrists' Autism Champion.

Psychiatrists in the UK play a critical role in the recognition, assessment and healthcare of patients on the autism spectrum. This is across a range of services (such as mental health, intellectual disability (also known as learning disability in UK health services), paediatrics) and in a variety of roles (for example triggering access to, or facilitating, a diagnostic pathway; making reasonable adjustments to enable access to services). As limited knowledge and awareness of autism is a key barrier to receiving appropriate diagnostic and therapeutic support,^1^ having psychiatrists with a strong understanding of autism is essential. Yet little is known about psychiatrists' knowledge about autism and about their confidence in making diagnostic or management decisions. The few existing studies – largely conducted outside of the UK – show that awareness of autism among professionals (for example psychiatrists, psychologists, neurologists, paediatricians, speech and language therapists) is variable^2–4^ and that there are substantial discrepancies in professionals' assessment and diagnostic practices.^5,6^ To our knowledge, the only UK-based studies to explore a range of professionals' views and experiences of working with children and adults on the autism spectrum^7,8^ focused exclusively on the diagnostic process, and comprised only few psychiatrists. There is, therefore, an urgent need to examine psychiatrists' experience of working with individuals on the autism spectrum, their confidence in doing so and the factors that affect these decisions.

## Method

### Online survey

Psychiatrists were invited to take part in an online survey open between September 2017 and January 2018. The survey was advertised via the Royal College of Psychiatrists, and internet snowballing methods, using social media, were used to recruit additional participants. The survey contained four parts and took approximately 15–20 min to complete.

Part one comprised 23 questions on the psychiatrists' background, including basic demographics (i.e. age, gender, ethnicity), details of their qualifications and experience (i.e. year qualified, country qualified in, years spent practising), current practice (i.e. geographic location, sector, role, speciality, length of time in this practice, patient contact hours/week), and information regarding training and experience related to autism (i.e. number of children and adults under their care, numbers of patients approaching them about an autism diagnosis, training on autism during and after qualifying as a psychiatrist, personal experience of autism).

Part two asked whether respondents were involved in the diagnosis of autism. If so, they were presented with eight questions on the use and utility of current diagnostic criteria, the procedures they would follow when patients do not meet formal diagnostic criteria or the threshold for autism on diagnostic tools, whether they followed a standardised procedure for autism diagnosis, the screening and diagnostic tools that they use (if any), and whether they have a waiting list for diagnostic assessments.

Part three comprised a Knowledge of Autism Scale, taken from a survey of UK-based general practitioners (GPs).^9^ This comprised 22 statements assessing participants' knowledge of the early signs of autism, descriptive characteristics of autism and co-occurring behaviours. Statements were rated as ‘true or false’. Participants were given a score of ‘1’ for each correct item and these scores were summed to yield a total score (with higher scores reflecting greater knowledge of autism; maximum score 22). A knowledge score was then calculated, adjusting for chance responding, using the following equation:
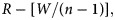


where *R* is the number of right responses, *W* is the number of wrong responses and *n* is the number of items.

The scale showed moderate internal consistency (Cronbach's α = 0.64) in line with other knowledge of autism scales.^9,10^

Part four was a self-efficacy scale, designed to assess respondents' confidence in the screening, diagnosis and management of their patients on the autism spectrum.^9^ This was adapted from an existing survey of GPs^9^ by removing items not applicable to psychiatrists (such as ‘Knowing to whom to refer my patients I suspect of having autism’) and adding additional items of relevance to psychiatrists (for example on knowledge of local care pathways and post-diagnostic services). The final survey contained 19 statements that respondents rated on a scale ranging from one (‘not at all confident’) to ten (‘extremely confident’). Scores from each item were averaged to yield a mean self-efficacy score (higher scores reflected greater self-efficacy). The scale showed excellent internal consistency (α = 0.96).

The survey ended with optional free-text boxes, asking respondents for their views on (a) their confidence in working with their autistic patients; (b) what they feel works well and what could be improved in this regard; (c) the training that they felt would help them more effectively work with their autistic patients; and (d) any other information they would like to add on the topic.

Ethical approval was granted by the Research Ethics Committee at UCL Institute of Education, University College London (REC 959). All participants provided informed consent to take part. Data were collected anonymously.

### Participants

In total, 229 psychiatrists responded to the online survey. Responses were not considered for those who were not currently practising psychiatrists in the UK (*n* = 50) or did not progress past the demographic information of the survey (*n* = 7). The final 172 respondents (Table 1) had a broadly even gender split, with an average age of 48 years and were largely from a White ethnic background (*n* = 120; 69.8%). Almost all had qualified as psychiatrists in the UK (*n* = 158; 91.9%) and the geographical distribution of their practice was diverse. Most worked in the public health sector (*n* = 153; 89.0%), as consultants (*n* = 159; 92.4%) in multidisciplinary teams (*n* = 165; 95.9%), and had a range of specialities. Respondents had spent an average of 19 years practising, with almost two-thirds (*n* = 106; 61.6%) spending more than 5 years in their current role. They reported an average of 19 patient-contact hours each week.

**Table 1:** Participant characteristics (*n* = 172)

Characteristic	Value
Age, years	
Mean (s.d.)	48.56 (8.93)
Range	31–73
Gender, *n* (%)	
Men	81 (47.1)
Women	90 (52.3)
Prefer not to say	1 (0.6)
Ethnicity, *n* (%)	
White	120 (69.8)
Black	4 (2.3)
Asian	33 (19.2)
Mixed	6 (3.5)
Any other ethnic group	2 (1.2)
Prefer not to say	7 (4.1)
Years practising as a psychiatrist, years	
Mean (s.d.)	18.60 (9.72)
Range	<1–48
Location of psychiatry practice, *n* (%)	
North East	11 (6.4)
North West	42 (24.4)
Yorkshire and the Humber	18 (10.5)
East Midlands	12 (7.0)
West Midlands	7 (4.1)
East of England	7 (4.1)
London	14 (8.1)
South East	14 (8.1)
South West	13 (7.6)
Wales	10 (5.8)
Scotland	21 (12.2)
Northern Ireland	3 (1.7)
Sector of work, *n* (%)	
Clinical, public	153 (89.0)
Clinical, private	2 (1.2)
Clinical, public and private	11 (6.4)
Non-clinical practice	–
Other	6 (3.5)
Role, *n* (%)	
Independent practitioner	6 (3.5)
Part of multidisciplinary team	165 (95.9)
Other	1 (0.6)
Consultant, *n* (%)	
Yes	159 (92.4)
No	13 (7.6)
Speciality, *n* (%)	
Addictions	4 (2.3)
Child and adolescent	40 (23.3)
Eating disorders	3 (1.7)
Forensic	4 (2.3)
General adult	59 (34.3)
Old age	12 (7.0)
Psychotherapy	2 (1.2)
Psychiatry of learning disabilities	29 (16.9)
Other	19 (11.0)
Practice, *n* (%)	
Community mental health team	86 (50.0)
Out-patient clinic	52 (30.2)
Hospital ward	43 (25.0)
General practice surgery	1 (0.6)
Other	34 (19.8)
Years in current role, *n* (%)	
<1	22 (12.8)
1–5	44 (25.6)
More than 5	106 (61.6)
Hours per week patient contact	
Mean (s.d.)	19.23 (8.60)
Range	2–41

### Data analysis

Quantitative data are largely presented descriptively (*n*, %), with statistical analyses used to examine group differences (Mann Whitney *U*-tests) and relationships between variables (using bivariate correlational or stepwise regression analyses). Participants' qualitative responses were analysed using thematic analysis.^11^ We adopted an inductive approach (i.e. without integrating the themes within any pre-existing coding schemes, or preconceptions of the researchers), within an essentialist framework (to report the experiences, meanings and reality of the participants). This involved three authors independently familiarising themselves with the data, and reviewing the transcripts to develop an initial set of themes and subthemes. The authors reviewed the results on several occasions, to resolve discrepancies and decide on the final themes and subthemes using a semantic approach (identifying themes at a ‘surface’ level, without theorising beyond the actual content of the data).

## Results

### Current practice when working with patients on the autism spectrum

Of the 172 respondents, just over a quarter reported having children on the autism spectrum under their care (*n* = 49; 28.5%). Of these, 38.8% (*n* = 19) had fewer than 10, 28.6% (*n* = 14) had between 11 and 30 and 32.7% (*n* = 16) had more than 30. A higher number (*n* = 120; 69.8%) reported having at least one autistic adult in their care. Of these, 55.0% (*n* = 66) had fewer than 10, 27.5% (*n* = 33) had between 11 and 30 and 17.5% (*n* = 21) had more than 30. In the past year, 86.6% (*n* = 149) of respondents had been approached by at least one patient about a suspected autism diagnosis, with 33.7% (*n* = 58) having been approached by more than ten. Most felt that this number had increased since beginning their professional career (*n* = 137; 79.7%), 19.2% (*n* = 33) felt it had remained steady, and 1.2% (*n* = 2) felt it had decreased.

### Training on autism

Just over two-thirds of respondents (*n* = 119; 69.2%) reported receiving specific training about autism during their primary medical degree, foundation degree or specialist psychiatric training (of these, 92 (77.3%) received this during specialist training). Many (*n* = 120, 69.8%) received specific training (for example via continuing professional development) on autism since qualifying as a psychiatrist. This included diagnostic training, on specific diagnostic tests such as the Autism Diagnostic Observation Schedule (ADOS),^12^ Autism Diagnostic Interview-Revised (ADI-R),^13^ Developmental, Dimensional and Diagnostic Interview^14^ and Diagnostic Interview for Social and Communication disorders (DISCO);^15^ but also intervention training (for example cognitive–behavioural therapy (CBT) for autistic patients) and generic autism awareness courses. A significant minority of respondents (30.8%, *n* = 53) reported that they received no training on autism during their primary medical or foundation degree or specialist psychiatric training. Of the 149 psychiatrists who had received some autism training, 76.5% (*n* = 114) reported that this was ‘quite’ or ‘very’ useful in preparing them to work with their autistic patients, with 19.5% (*n* = 29) reporting that the training was ‘not very’ useful and 4.0% (*n* = 6) reporting that it was ‘not at all’ useful.

### Personal experience of autism

A total of 81 respondents (47.1%) reported some personal experience of autism, either through being autistic themselves (*n* = 2), having an autistic child (*n* = 12) or a relative (*n* = 36), colleague or friend (*n* = 32) on the autism spectrum.

### Knowledge of autism

A total of 162 respondents completed the Knowledge of Autism Scale, scoring highly (mean 91.2% correct; s.d. = 9.3, range 31.8–100.0%) (Table 2). Respondents' scaled knowledge scores expressed as a percentage of the total number of questions asked (mean 90.6%; s.d. = 9.7; range = 28.6–100.0%) were not significantly associated with their age, *r*(162) = 0.04, *P* = 0.64, number of years practising as a psychiatrist, *r*(162) = 0.02, *P* = 0.76 or total number of autistic patients currently under their care, *r*(162) = 0.05, *P* = 0.50. Mann–Whitney *U*-tests showed that scaled knowledge scores were significantly higher for psychiatrists with personal experience of autism (median 20.95) that those without (median 19.90), *U* = 2570, *P* = 0.02; but there was no significant difference in scores between psychiatrists who had received training on autism and those who had not, *U* = 1302.5, *P* = 0.70.

**Table 2:** Psychiatrists correct responses to items on the Knowledge of Autism Scale (*n* = 162)

Item	Answer (true/false)	Psychiatrists giving a correct response, *n* (%)
An autism diagnosis cannot be made before a child is 3 years of age	False	99 (61.1)
A child failing to respond to their name when called can be an early sign of autism	True	118 (72.8)
A lack of eye contact is necessary for a person to receive a diagnosis of autism	False	157 (96.9)
Research has shown that the measles, mumps, rubella vaccine is not a direct cause of autism	True	153 (94.4)
Autism is caused by a lack of bonding between mother and child	False	159 (98.1)
Autism is a rare condition, affecting only 15 per 10 000 individuals in the UK	False	150 (92.6)
Autism cannot be diagnosed in adulthood	False	161 (99.4)
The behaviours characteristic of autism are usually mild and transient, so specific intervention is not usually required	False	156 (96.3)
The prevalence of autism is greater in children than in adults	False	131 (80.9)
Younger siblings of children with autism have a higher probability (approximately 20%) of being diagnosed with autism	True	142 (87.7)
Most people with autism also have intellectual disabilities	False	113 (69.8)
Females are less likely to be diagnosed with autism than males	True	155 (95.7)
People with autism feel no empathy or affection	False	156 (96.3)
Children with autism can be interested in social interaction	True	153 (94.4)
More than half of people diagnosed with autism do not talk	False	151 (93.2)
Children with autism can show unusual reactions to certain smells and sounds	True	160 (98.8)
Additional mental health conditions (for example, anxiety, depression) are more prevalent in individuals diagnosed with autism than in the general population	True	158 (97.5)
Most children with autism eventually outgrow autism	False	159 (98.1)
Independent living is not possible for people with autism	False	161 (99.4)
The behaviours in autism can only be managed with medication	False	161 (99.4)
People with autism always display challenging behaviours	False	156 (96.3)
Children with autism tend to learn better when things are presented in a visual format	True	135 (83.3)

### Diagnosing autism – processes and pathways

A total of 111 psychiatrists reported that they were involved in diagnosing patients on the autism spectrum, and 109 provided information about waiting times: 55 (50.5%) reported having a waiting list for autism diagnostic assessments and, of these, 21.8% (*n* = 12) had an average wait time of 1–3 months and 78.2% (*n* = 43) had an average waiting time of over 3 months.

Of the 111 psychiatrists involved in diagnosis, the majority (*n* = 89; 80.2%) reported that they would refer to ICD^16^ diagnostic criteria when considering possible autism, and 56.8% (*n* = 63) reported referring to DSM^17,18^ criteria. Most psychiatrists (*n* = 98; 88.3%) found these diagnostic criteria ‘somewhat’, ‘very’ or ‘extremely’ useful when making diagnostic decisions (only 13 psychiatrists (11.7%) found them ‘a little’ or ‘not at all’ useful).

Of the 110 psychiatrists involved in autism diagnosis, 69.1% (*n* = 76) had experienced situations where a child or adult did not meet the formal diagnostic criteria or threshold for autism on diagnostic tools, but their clinical judgement suggested otherwise (for example those showing atypical presentations of autism, such as women and girls on the autism spectrum). Of these, 36.8% (*n* = 28) reported that they would give the individual a diagnosis of autism in this instance. The remainder provided alternative options, for example: offering an alternative diagnosis (such as autistic traits or features); using additional diagnostic tools to confirm their diagnoses; or referring the patient to an autism specialist team. They also considered the utility of the diagnosis for the individual and their family when deciding whether to give an autism diagnosis.

In total, 111 of the psychiatrists involved in diagnosing autism answered questions about their protocol for autism diagnosis and the measures they use. Of these, 78.4% (*n* = 87) reported following a standardised protocol for autism diagnosis, often drawn from commonly used diagnostic instruments (such as the ADOS, ADI-R) and local and/or national guidance.^19,20^ Psychiatrists also reported using clinical judgement (*n* = 78; 70.3%), clinical observations (*n* = 71; 64.0%), parent report (*n* = 66; 59.5%), standardised structured interview (*n* = 38; 34.2%), standardised observation measures (*n* = 26; 23.4%) and DSM or ICD diagnostic criteria (*n* = 61; 55.0%). 44.1% of psychiatrists (*n* = 49) reported using a combination of all of the above to inform their diagnosis.

The most commonly used screening measure was the freely available Autism Quotient^21^ (*n* = 43; 38.7%), with a wide number of other screening tools (including the Social Communication Questionnaire,^22^ the Ritvo Autism Asperger Diagnostic Scale – Revised,^23^ the Social Responsiveness Scale^24^ and the Autism Behaviour Checklist^25^ being infrequently used. Almost half of the psychiatrists (*n* = 51; 45.9%) reported routinely using the ADOS-2, making this the most commonly used diagnostic measure, followed by the ADI-R (*n* = 41; 36.9%) and the DISCO (*n* = 20; 18.0%). When assessing patients potentially on the autism spectrum, psychiatrists often reported using a range of other assessments, including those indexing co-occurring psychiatric conditions (*n* = 76; 68.5%), co-occurring neurodevelopmental conditions (*n* = 62; 55.9%), functional ability (*n* = 62; 55.9%), medical problems (*n* = 59; 53.2%), cognitive ability (such as intelligence tests; *n* = 39; 35.1%) and mental capacity (*n* = 32; 28.8%). A total of 16.2% of psychiatrists (*n* = 18) who were involved in autism diagnosis reported using all of these additional assessments, with 9% (*n* = 10) not using any formal assessment.

### Self-efficacy

Respondents' confidence in their ability to screen, diagnose and manage their patients on the autism spectrum ranged widely (Table 3). Correlational analyses showed that higher self-efficacy scores were significantly related to better scaled knowledge of autism scores, *r*(158) = 0.26, *P* = 0.001 and the total number of autistic patients currently in their care, *r*(159) = 0.44, *P* < 0.001. Self-efficacy scores were not related to years practising as a psychiatrist, *r*(159) = −0.005, *P* = 0.95. Mann–Whitney *U*-tests revealed that self-efficacy scores were significantly higher for psychiatrists who had received training on autism (median 7.05) compared with those who had not (median 4.68), *U* = 523, *P* < 0.001; yet there was no significant difference in self-efficacy scores between psychiatrists who had personal experience of autism and those who did not, *U* = 2833, *P* = 0.17.

**Table 3:** Mean and mode scores for each item on the self-efficacy scale^a^

Item	Mean score (s.d.)	Range	Mode score
Recognising the signs and symptoms of autism in children	5.87 (2.70)	1–10	5.00
Recognising the signs and symptoms of autism in adults	6.83 (1.80)	1–10	6.00
Recognising the signs and symptoms of autism in boys and men	7.10 (1.92)	1–10	8.00
Recognising the signs and symptoms of autism in girls and women	6.38 (1.94)	1–10	7.00
Recognising the signs and symptoms in individuals with good spoken language and no apparent intellectual difficulties	6.73 (2.12)	1–10	8.00
Recognising additional psychiatric disorders (e.g. anxiety, depression) in autistic people who access my service	7.61 (1.80)	2–10	8.00
Managing additional psychiatric disorders (e.g. anxiety, depression) in autistic people who access my service	7.22 (2.10)	1–10	8.00
Recognising additional neurodevelopmental conditions (e.g. ADHD, OCD) in autistic people who access my service	6.94 (2.15)	1–10	9.00
Managing additional neurodevelopmental conditions (e.g. ADHD, OCD) in autistic people who access my service	6.60 (2.29)	1–10	8.00
Contributing effectively to helping services to manage the care of children on the autism spectrum	5.07 (2.94)	1–10	1.00
Contributing effectively to helping services manage the care of adults on the autism spectrum	5.66 (2.46)	1–10	5.00
Assessing the strengths, needs and aspirations of autistic people who access my service	6.48 (2.30)	1–10	8.00
Communicating with patients about a suspected diagnosis of autism	6.92 (2.21)	1–10	8.00
Knowing to whom to refer my patients I suspect of having autism	7.43 (2.55)	1–10	10.00
Knowing local care pathways for patients to access diagnostic assessments	7.43 (2.75)	1–10	10.00
Knowing local post-diagnostic services to refer patients who have received an autism spectrum diagnosis	6.13 (2.93)	1–10	8.00
Knowing the relevant care pathways/services for people on the autism spectrum	6.16 (2.76)	1–10	8.00
Knowing which community resources in my area are available for autistic children and/or adults	5.94 (2.72)	1–10	8.00
Identifying stress in the parents and carers of autistic people who access my service	7.00 (2.22)	1–10	8.00
Total	6.35 (2.43)		

ADHD, attention–deficit hyperactivity disorder; OCD, obsessive–compulsive disorder.

a. Scores range from 1 (‘not at all confident’) to 10 (‘extremely confident’).

### Predicting psychiatrists' self-efficacy

Multiple regression analysis was used to predict psychiatrists' perceived self-efficacy, with years spent practising as a psychiatrist, the number of autistic patients currently under their care, training on autism (either during their initial training or later in their careers) and personal experience of autism entered stepwise into the model, together with scaled knowledge scores. As shown in Table 4, respondents' total number of autistic patients currently under their care as well as autism training were significant predictors, together explaining 24% of the variance in psychiatrists' self-efficacy scores. There were no other significant predictors (all *P*s > 0.07), final model: *F*(2, 159) = 23.04, *P* < 0.001.

**Table 4:** Summary of hierarchical regression analysis predicting psychiatrists' self-efficacy scores^a^

	*b* (s.e.)	*β*
Step 1		
Constant	5.36 (0.29)	–
Number of patients on the autism spectrum currently under their care	0.28 (0.06)	0.37*
Step 2		
Constant	3.77 (0.46)	–
Number of patients on the autism spectrum currently under their care	0.27 (0.05)	0.36*
Autism training	1.83 (0.42)	0.30*

a. *R*^2^ = 0.14 for Step 1, Δ*R*^2^ = 0.09 for Step 2 (*Ps* < 0.001).

* *P* < 0.001.

### Qualitative analysis of open-ended responses

**Fig. 1:**
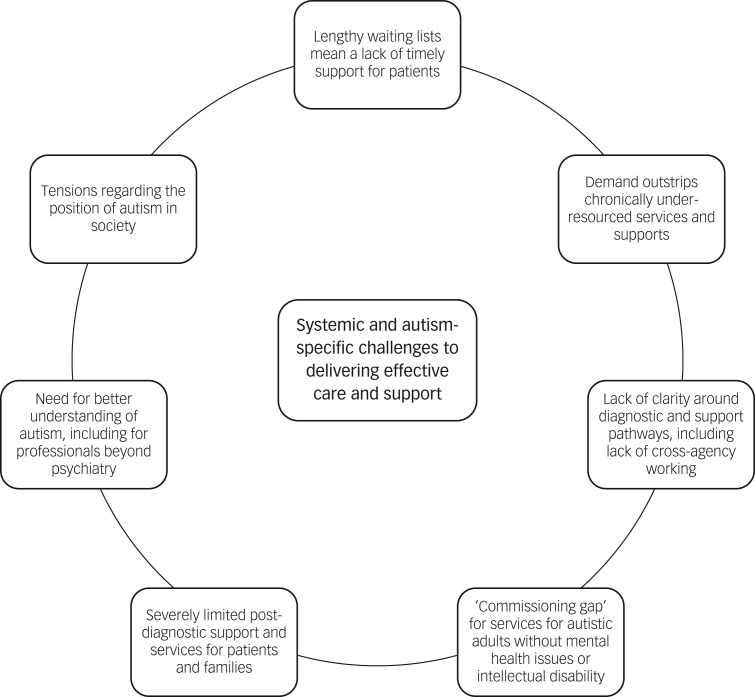
The seven themes identified, which describe a range of systemic and autism-specific challenges to delivering the most effective care and support.

Psychiatrists reported that working with their autistic patients was ‘very, very rewarding’ work, and that ‘making a good assessment and providing appropriate psychoeducation can make great difference to the lives of these families’. Another commented that ‘the variety is immense’ and working with autistic patients offers ‘an insight into a completely different way of looking at the world, it is fascinating’. Despite these positive sentiments, we identified seven themes, describing a range of systemic and autism-specific challenges to delivering the most effective care and support (Fig. 1).

#### Lengthy waiting lists mean a lack of timely support for patients

Psychiatrists expressed concern that lengthy waiting lists mean a lack of timely support for patients, with many speaking of waiting periods during which the patient was not receiving any help or support: ‘we need timely access to diagnostic services. Currently we have a 1-year wait for assessment and during that 1-year wait we are lost as to how to help most persons’. One respondent referred to these waiting times as ‘unacceptably long’ and emphasised a need for ‘more personnel involved in multiagency assessment, identification and management’.

#### Demand outstrips existing, chronically underresourced services and supports

In line with these comments, psychiatrists reported that demand outstrips existing, chronically underresourced services and supports, highlighting a broader issue in terms of healthcare resources. As one respondent noted: ‘services for general adult psychiatry are woefully underprovided – autism is low on the pecking order. There is no plan for service provision in general, never mind autism support’. Many reported frustration over the lack of staff at all levels in their services, which they felt ‘affects wait times and consistency for this group of patients’, as their time is ‘spent on tasks that other [administrative] staff could do’. Psychiatrists stressed that this had seriously negatively effects on the care they could provide as they ‘never [have] enough resources for patients who should be getting help’. These generic problems were felt to be compounded for autistic patients as the diagnostic process is often lengthy: ‘[we need] more time and resources to assess these people, as it is very time consuming to do properly’. Further, as a result of increased recognition, ‘autism cases are rocketing’ and ‘whilst increased recognition and understanding is helpful, this has increased demand on services that already cannot cope’. One psychiatrist explained: ‘we are getting three referrals specifically for autism diagnosis or management per week… it's all chaos’.

#### Lack of clarity around diagnostic and support pathways, including lack of cross-agency working

Psychiatrists further complained of a lack of clarity around diagnostic and support pathways, including lack of cross-agency working, expressing concern that there is ‘no local guidance for diagnosis or management of autism and no agreed screening or assessment pathways’. For many, this meant that services were ‘very poorly signposted and confusing’, and ‘sometimes difficult to access’. Psychiatrists noted that ‘working effectively with other agencies presents major challenges’ and stressed the need for a ‘more joined up approach between social care, education and medical colleagues’; currently, they felt a ‘big gap’ exists, ‘with many children falling in between… and therefore not getting the help that they need’. Some psychiatrists touched upon the idea of developing a ‘specialist service’ for autism diagnosis and support which ‘understands their needs’ and could ‘serve patients best’. Yet, others argued that ‘fragmented commissioning is not helpful’ suggesting that autism services should be ‘within the mainstream services of adult mental health and not in isolation’ because of co-occurring developmental and mental health conditions.

#### ‘Commissioning gap’ for services for autistic adults without mental health issues or intellectual disability

Psychiatrists identified a ‘commissioning gap’ for services for autistic adults without mental health issues or intellectual disability. They felt that provision of services for these autistic adults and young people was ‘markedly lacking’, with one commenting that ‘some see [individuals without a learning disability or mental health comorbidity] as not the remit of psychiatrists’. This meant that those ‘without significant social care needs or psychiatric comorbidity [were] left without any support’ – they may be seen as ‘higher functioning’ and as such are not ‘important enough’, so do not receive the support and services they require. Despite services being hard to access without additional difficulties, psychiatrists also reported significant issues meeting the needs of those with these difficulties, commenting that mental health services are ‘reluctant to work with autistic individuals’. They highlighted the lack of ‘co-ordinated mental health services’ for those with autism and mental health problems, noting that such patients ‘can often be highly challenging to work with and staff are not trained appropriately’. As a result, there was uncertainty as to who could help them: ‘the local autism team provides social care only and are hesitant to joint work if there is a potential mental health issue’. Meanwhile, there were a ‘lack of therapists who have experience and understanding of how to adapt CBT for young people and adults with ASC [autism spectrum condition] and depression/anxiety’. The general sentiment was that there are ‘few professionals who feel confident managing [mental health difficulties] in autism and no provision for those who show difficult behaviours’, resulting in them ‘apologising a lot’. The respondents called for ‘greater acceptance’ and ‘greater overall knowledge’ of autism in mental health services and ‘improved recognition [and] intervention for mental health comorbidities’ across the board.

#### Severely limited post-diagnostic support and services for patients and their families

These commissioning gaps contributed to a general lack of support more broadly, with many psychiatrists raising the severely limited post-diagnostic support and services for patients and their families as a key issue. Psychiatrists reported being ‘concerned’ and ‘frustrated’ by the lack of post-diagnostic services in place, with one commenting: ‘At the moment, people can be assessed for autism, but that's where the road ends.’ Even if some services exist, another psychiatrist observed: *‘*I have knowledge of the limited options for post-diagnostic support that exist in my locality. The difficulty is that there are not enough (capacity or range) options available’. As a result of the lack of National Health Service (NHS) follow-up services available, some psychiatrists reported turning to charities to help, however, they recognised that ‘there are limited funds to support [charitable] organisations’ so they could only help a small number. Respondents called for more investment and resources for post-diagnostic support services so that individuals have ‘easier access to support across the lifespan’, both within the health service and for ‘better community support’ so that autistic individuals could ‘integrate properly into mainstream schools’ and receive help ‘entering employment’. The lack of post-diagnostic support may exacerbate the constraints to care already noted: ‘The lack of access to specific services for people with autism has caused repeated readmissions in many of my patients. They do not have the right support in the community’.

#### Need for better understanding of autism, including for professionals beyond psychiatry

Many psychiatrists emphasised the need for better understanding of autism, including for professionals beyond psychiatry. They stressed that there needed to be ‘greater specialist training for all multidisciplinary team staff [as] generally confidence is low with autism cases’ and that ‘better general awareness’ was needed in ‘all professional groups’. One psychiatrist commented that colleagues seemed ‘very reluctant or ignorant in dealing with people with autism’, and another found that ‘colleagues both medical and non-medical [are] relatively unsympathetic to patients with the condition and have difficulty grasping concepts’. The respondents highlighted that ‘colleagues and trainees who haven't had adequate clinical exposure particularly to “typical cases” may misunderstand what is meant by some of the features’, and that this ‘lack of understanding’ could lead to misdiagnosis or failure to recognise ‘various comorbid difficulties which often overlap with ASC’. Overall this can result in ‘challenges in delivering a satisfactory service’ to autistic individuals, thus they called for more training ‘all round’ to improve patient care.

Psychiatrists who were more confident recognised that personal and professional experience helps. Psychiatrists who had regularly seen autistic individuals as part of their ‘day-to-day’ job were more confident supporting them. One psychiatrist recognised that because of their experience, they were ‘certainly more [confident] than [they] would have been earlier in [their] work career’. A few psychiatrists reported that they ‘undertook specific training’ as they recognised a need for autism awareness in their professional practice: ‘I have had a special interest in the condition as I was aware the diagnosis may have been missed in several patients on my case load’. Others attributed their knowledge to personal experience of autism, for example having a child on the autism spectrum: ‘A lot of my knowledge comes from carrying out additional reading and study due to having a child with autism myself, rather than through what I learnt during training’.

#### Tensions regarding the position of autism in society

Psychiatrists reported tensions regarding the position of autism in society, and whether it should be viewed as a disability or a difference. As summarised by one respondent: ‘autism, like some other diagnostic entities, occupies an interesting position in society, in that it is defined in the diagnostic manuals as having areas of impairment (akin to deficit model), yet for some autistic individuals this is/feels oppressive and disabling/disablist’. This poses a difficult balance for psychiatrists as they ‘look hard for the things that children cannot do and the social skills that are missing’; however, this can result in diagnoses being ‘disabling’ as parents have lower expectations of what their child is able to do (‘he can't do that, he's autistic’). Some therefore stressed a need to ‘ensure that we speak about the positives and strengths of young people with autism’. For one psychiatrist, it was unclear whether diagnosis was actually ‘necessary or helpful in all cases’ considering some people have ‘developed strategies to enable them to function well without distress’. However, another highlighted that ‘those with LD [learning disability] and autism may be unintentionally excluded’ by this as ‘they go against growing sentiment that autism cannot or should not be characterised by overt difficulty/disability’, making it hard to strike a fair balance that meets the needs of this very heterogeneous group without being overly pathologising in the process.

## Discussion

### Main findings

The psychiatrists who participated in this study reported that they commonly encountered patients on the autism spectrum as part of their professional roles, and acknowledged that the number of autistic patients on their case-loads was increasing. This finding demonstrates the importance and timeliness of the current survey, which – to our knowledge – is the first to exclusively survey psychiatrists about autism. Psychiatrists reported working with patients on the autism spectrum to be a rewarding part of their role. Most had received training on autism, which they found to be useful, and their knowledge about autism was high, particularly for those with a personal connection to autism. Psychiatrists' self-efficacy varied in relation to different aspects of their role, but higher levels of self-efficacy were linked to greater knowledge, experience and training in autism. Analyses of open-ended data highlighted a number of systemic and autism-specific factors that psychiatrists felt challenged their ability to provide the most effective care and support for their autistic patients.

With high levels of knowledge, experience and training in autism (all of which were related to increased self-efficacy), the psychiatrists surveyed in this study had good knowledge of diagnostic tools and processes, and used these in conjunction with clinical judgement to best meet the needs of their patients. Notwithstanding, they reported several systemic factors (largely perceived to be outside of their control) that challenged their ability to work effectively with their autistic patients. In relation to autism diagnosis – a key area in which psychiatrists are involved – the current findings echo those reports from parents^8,26^ and autistic adults^1,26^ in highlighting lengthy waiting times and limited post-diagnostic support for children and adults on the autism spectrum. They also confirm the lack of clarity regarding diagnostic pathways for patients on the autism spectrum, as reported by other UK professionals involved in the autism diagnostic process.^7,9^

### Initiatives to improve support and services

The Royal College of Psychiatrists has implemented several measures to begin to address such issues. For example, the Royal College of Psychiatrists recently worked with the Department of Health, NHS Digital and other partners to ensure that autism was more prominent in the Mental Health Minimum Data Set for England (collecting patient-level data on children, young people and adults in contact with mental health, intellectual disabilities or autism services). Likewise, since April 2018, data have been collected on autism diagnosis waiting times. The goal is that these initiatives will aid in service and delivery planning and start to address unwarranted variance in autism support and services across England. The current findings will further inform these and future Royal College of Psychiatrists initiatives to help improve psychiatrists' confidence in working with their autistic patients.

### Co-occurring conditions

Our sample of psychiatrists highlighted specific challenges meeting the needs of currently underserved groups of the population on the autism spectrum, such as autistic adults with co-occurring mental health conditions and/or intellectual disabilities. They noted that both mental health services and autism-specific services were reluctant to work with autistic patients with mental health problems. These reports corroborate those of autistic adults, who have voiced their concerns at the lack of clear pathways when seeking help for their mental health problems.^27^ As up to 70–80% of people on the autistic spectrum have additional psychiatric diagnoses,^28,29^ and autistic adults without intellectual disabilities are nine times as likely to die by suicide,^30^ it is essential that services are well-equipped to support autistic people who have co-occurring mental health diagnoses. Together, these findings will inform the Royal College of Psychiatrists' initiatives to ensure that autism does not become a diagnosis of exclusion and that mental health services are able to make person-centred reasonable adjustments to ensure autistic people who have mental health conditions have equity of access and service with non-autistic people.

### The issue of specialist services

One further tension in the current study focused on whether there should be specialist services for patients on the autism spectrum, or whether existing services should be more inclusive of autistic patients. Although our sample of psychiatrists often discussed these possibilities as a dichotomy, it may be that the adoption of both models of service delivery could be advantageous. For example, specialist diagnostic assessment may be helpful to identify the specific strengths, needs and aspirations of the patient, provide initial post-diagnostic support and serve as a consultation or liaison model to primary care services. Yet, such specialist services should not prevent autistic people from being able to access generic primary care services, as well as specialist physical health, mental health or intellectual disability services, without any more barriers or restrictions than those that apply to people who are not autistic. Whatever the model of service delivery, there will be the need to make reasonable adjustments to ensure that access for autistic patients is both meaningful and equitable. One key way to achieve this is to involve members of the autism community in service planning, to ensure that the resulting services are respectful, accessible and patient-centred.^9,31^

### Limitations

This survey represents the first to focus exclusively on psychiatrists' knowledge, experience and confidence in working with their patients on the autism spectrum. It is not, however, without its limitations. First, although 28.5% of respondents had children on the autism spectrum on their case-load, the majority (69.8%) had autistic adults as their patients, which may have had an impact on some of the findings of the survey (for example regarding their reports of a relative lack of confidence in supporting children on the autism spectrum). Second, while the sample represented a high number of psychiatrists relative to other research that has included this professional group,^7,26^ the response rate was fairly low: with an estimate of around 7000 psychiatrists practising in the UK,^32^ approximately 2.5% participated in this survey. Third, given that just under half of respondents reported having some personal connection with autism (as found in research on UK GPs),^9^ the sample may also be biased, with those with a keen interest in autism being more likely to respond. This limitation suggests that we must exert caution in interpreting these results but also, critically, that we may be underestimating some of the issues at hand.

With autism now listed as an NHS priority in England, and with a Royal College of Psychiatrists Championing Autism campaign currently running to July 2020, there is an opportunity to enact meaningful changes to the way psychiatry supports children, young people and autistic adults in England. Investment in more effective – and particularly, local – services should contribute to a reduced need for interactions with psychiatric services, especially in times of crisis, and ultimately serve to improve the mental well-being of people on the autism spectrum and their families.
